# Piperacillin/Tazobactam‐Induced Bilateral Optic Disc Edema With Serous Retinal Fluid in a Young Female With Pelvic Abscess and Sepsis

**DOI:** 10.1155/crdi/2925958

**Published:** 2026-07-13

**Authors:** Georgios Papaetis, Ioannis Kazakos, Eleftheria Lazarou, Pantelis Kountourakis, Charis Antoniou, Konstantinos Mikellidis

**Affiliations:** ^1^ Internal Medicine and Diabetes Clinic, K.M.P. Therapis Paphos Medical Center, 14 Vasileos Georgiou B Street Office 201, Paphos, 8010, Cyprus; ^2^ CDA College, 73 Democratias Avenue, Paphos, Cyprus; ^3^ Obstetrics and Gynecology Clinic, Ygia Polyclinic Private Hospital, 21 Nafpliou Street, Limassol, 3025, Cyprus; ^4^ Department of Obstetrics, Iasis Private Hospital, Paphos, Cyprus; ^5^ Department of Nursing, School of Health Sciences, University of Technology, Limassol, Cyprus, ltu.se; ^6^ Medical Oncology Department, Mediterranean Hospital, 9 Stygos Street, Limassol, 3117, Cyprus; ^7^ Paphos Eye & Laser Clinic, Saint George Private Hospital, Eleftheriou Venizelou 29, Paphos, 8021, Cyprus; ^8^ Obstetrics and Gynecology Clinic, K.M.P. Therapis Paphos Medical Center, 14 Vasileos Georgiou B Street Office 201, Paphos, 8010, Cyprus

**Keywords:** optic disc edema, optical coherence tomography, piperacillin/tazobactam, sepsis, serous retinal fluid

## Abstract

**Introduction:**

The typical visual symptoms of optic disc edema are transient visual obscurations due to fluctuations of the optic nerve head blood perfusion. If it remains untreated, it can cause damage and significant vision loss. Piperacillin/tazobactam (PIP/TAZ) exerts broad activity against most gram‐positive and gram‐negative aerobic and anaerobic bacteria, including many pathogens producing beta‐lactamases. It is administered for the treatment of moderate‐to‐severe bacterial infections. Ocular adverse reactions after the administration of PIP/TAZ have been rarely reported.

**Case Presentation:**

We report a case of a 32‐year‐old woman, who developed pelvic abscess and sepsis, as a complication of caesarean section. She experienced bilateral optic disc edema with associated intraretinal and subretinal fluid during PIP/TAZ therapy. PIP/TAZ was discontinued the fourth day of its administration and was substituted with ertapenem. Progressive resolution of optic disc edema in both eyes, with complete normalization of the fundus and retinal nerve fiber layer thickness, was observed 2 months after drug discontinuation. However, persistent bilateral discontinuity of the foveal ellipsoid zone remained.

**Conclusion:**

Bilateral optic disc edema with serous retinal fluid after PIP/TAZ therapy is very rare. Prompt discontinuation of the medication and ophthalmic evaluation should be immediately considered when visual symptoms arise. Further pharmacovigilance reporting is warranted to clarify the rarity of this adverse effect after PIP/TAZ administration.

## 1. Introduction

Optic disc edema is mainly caused by ischemia, inflammation, or increased intracranial pressure (ICP‐papilledema) [1, 2]. The typical visual symptoms of optic disc edema are transient visual obscurations due to temporary fluctuations of the optic nerve head blood perfusion [3, 4]. If it remains untreated, it can cause damage and significant vision loss [3–5]. Optic disc edema is usually bilateral and symmetric, but it can be asymmetric or unilateral. Its main causes are (i) intracranial neoplasms, (ii) subarachnoid hemorrhage and subdural hematoma, (iii) idiopathic intracranial hypertension (pseudotumor cerebri), (iv) intracranial inflammation and/or infection, (v) vascular causes such as cerebral venous sinus thrombosis, (vi) head trauma, (vii) malignant hypertension, (viii) drug induced, (ix) nutritional deficiencies, (x) obstructive/noncommunicating hydrocephalus, and (xi) idiopathic [1, 2, 6–9].

Drug‐induced optic disc edema is usually bilateral and presents with vision changes and headaches. The most common causative drugs that have been associated with optic disc edema are corticosteroids (prolonged therapy or withdrawal); antibiotics (such as sulfa‐containing antibiotics, minocycline, doxycycline, nitrofurantoin, and nalidixic acid); vitamin A, isotretinoin, and hormones (such as growth hormone, LHRH analogs, and levonorgestrel), chemotherapy (such as ponatinib, oxaliplatin, and carboplatin), cyclosporin, lithium, mirtazapine, fluvoxamine, sertraline, and phenytoin [10–14]. We report a case of a 32‐year‐old woman, who developed pelvic abscess and sepsis as a complication of caesarean section. She experienced bilateral optic disc edema with associated intraretinal and subretinal fluid during piperacillin/tazobactam (PIP/TAZ) therapy.

## 2. Case Presentation

A 32‐year‐old Cypriot woman presented to our clinic during the early puerperium after she delivered her third child via cesarean section. She reported progressive lower abdominal pain, abdominal distension, and dysuria during the last 2 weeks. One week before her presentation, she was treated with a 7‐day course of amoxicillin/clavulanic acid, as a presumed urinary infection, from her general practitioner. Her urine analysis showed 4‐5 leukocytes/high‐power field, while urine cultures were negative before starting the antibiotic therapy. Her past medical history was significant for two prior cesarean sections (2021 and 2024). She did not report any hypersensitivity reactions, and she had not been prescribed any medications systematically in the past. No past ocular history was reported.

Her physical examination showed that she experienced all three quick sepsis‐related organ failure assessment (SOFA) criteria: (i) altered mentation (Glasgow Coma Scale score: 13), (ii) respiration rate of 30 breaths/minute, and (iii) systolic blood pressure of 100 mmHg [15]. Her body temperature was 38°C, and her heart rate was 110 beats/minute. Physical examination revealed marked abdominal tenderness, involuntary guarding and rebound tenderness suggesting peritoneal irritation. The rest of the clinical examination was unremarkable. Results of her laboratory investigations showed mild anemia (hemoglobulin: 11 g/dL) and white blood cell count of 18.200/mm^3^ (neutrophils: 82%). The erythrocyte sedimentation rate was 80 mm/h, and the C‐reactive protein level was 25.3 mg/dL. Other laboratory investigations were within normal limits, including liver and renal function indices, electrolytes, and urine analysis/culture.

**FIGURE 1:**
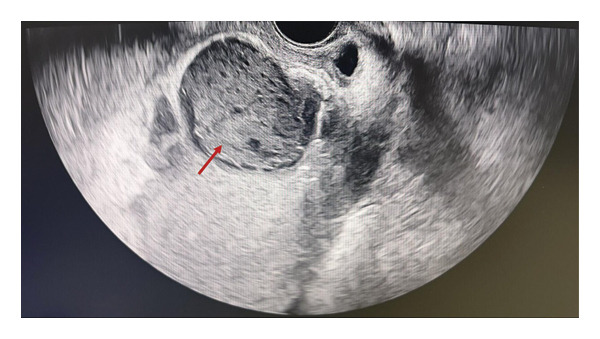
Transvaginal ultrasonography during her presentation. Pelvic fluid collection measuring 55 × 35 mm (red arrow) was found between the uterus and the urinary bladder.

Transvaginal ultrasonography demonstrated a pelvic fluid collection measuring 55 × 35 mm located between the uterus and the urinary bladder (Figure 1). Contrast‐enhanced abdominal computed tomography confirmed the pathological collection and was highly suspicious for pelvic abscess with associated peritonitis. After her admission to the hospital, PIP/TAZ was introduced in a daily dose of 4.5 g every 6 h. An exploratory laparotomy was performed during the same day. A localized pelvic abscess was identified intraoperatively and was surgically drained, followed by thorough peritoneal lavage and placement of an appropriate drainage. Strains of extended‐spectrum beta‐lactamase producing *Escherichia coli* and *Bacteroides fragilis* were isolated from the cultures of the abscess. Both pathogens were sensitive to PIP/TAZ.

During the third day of PIP/TAZ therapy, the patient reported short episodes of gray‐out, blurred vision, which deteriorated significantly during the next day. Given the possibility that PIP/TAZ was the cause of her ocular symptoms, it was discontinued on the fourth day of her hospitalization and was substituted with ertapenem 1 g/daily. The patient’s ocular symptoms started to improve 24 h after the discontinuation of PIP/TAZ. She was also immediately referred for ophthalmic evaluation. Initially, the patient showed reduced visual acuity (best corrected visual acuity in both eyes [OU BCVA: 6/12]), normal intraocular pressure, and unremarkable anterior segment. Fundoscopic examination revealed bilateral stage III optic disc edema. Optical coherence tomography (OCT) demonstrated marked bilateral retinal nerve fiber layer (RNFL) elevation and bilateral intraretinal and subfoveal fluid. The patient underwent magnetic resonance imaging (MRI) of the brain, which suggested only slight bilateral globe prolapse. She was advised to undergo a lumbar puncture (LP) in order to measure cerebrospinal fluid opening pressure (CSFOP). The patient consistently refused to undergo this procedure. She was discharged after a 10‐day course of ertapenem with complete remission of the infection.

**FIGURE 2:**
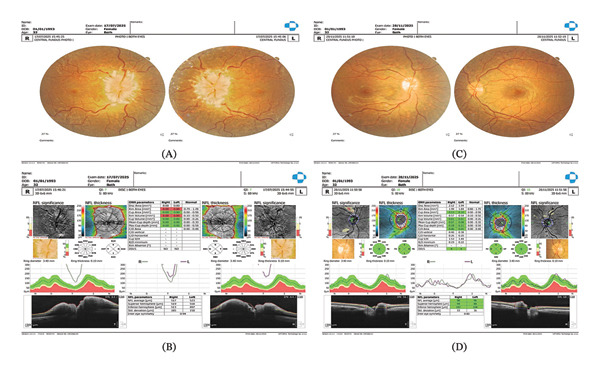
Initial ophthalmic evaluation during the fourth day of PIP/TAZ therapy (A): Bilateral optic disc edema stage III; (B): bilateral remarkable RNFL elevation shown on OCT‐ONH scan. Ophthalmic evaluation 2 months after PIP/TAZ discontinuation (C): normal‐looking fundus; (D): RNFL thickness normalizing shown on OCT‐ONH scan. PIP/TAZ: piperacillin/tazobactam; ONH: optic nerve head; RNFL: retinal nerve fiber layer; OCT‐ONH: optical coherence tomography of the ONH.

**FIGURE 3:**
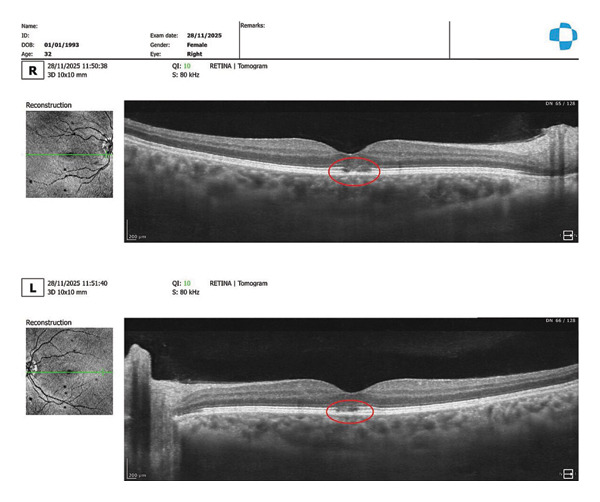
Residual foveal EZ changes (red circle) 2 months after PIP/TAZ discontinuation. PIP/TAZ: piperacillin/tazobactam; EZ: ellipsoid zone.

**FIGURE 4:**
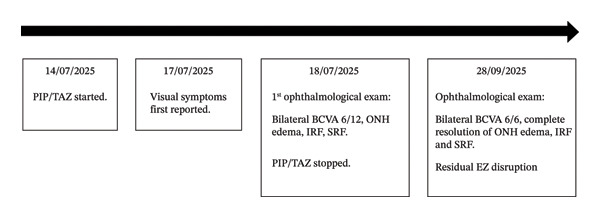
Timeline from diagnosis to discharge and follow‐up. PIP/TAZ: piperacillin/tazobactam; ONH: optic nerve head; BCVA: best corrected visual acuity; IRF: intraretinal fluid; SRF: subfoveal fluid; EZ: ellipsoid zone.

During the first month of follow‐up, visual acuity improved to 6/6 in both eyes. Optic disc edema and RNFL thickening were markedly decreased and retinal fluid was resolved, although foveal ellipsoid zone (EZ) disruptions were noted. During subsequent follow‐up visits, she demonstrated continued bilateral improvement, with sustained normal visual acuity and stable intraocular pressure. Progressive resolution of bilateral optic disc edema, with complete normalization of the fundus and RNFL thickness, was observed 2 months after PIP/TAZ discontinuation. Retinal OCT consistently showed no recurrence of intraretinal or subfoveal fluid. Visual field sensitivity ultimately returned to normal levels in both eyes. However, bilateral discontinuity of the foveal EZ remained. Fundus pathology and OCT scans 4 days after PIP/TAZ administration and 2 months after its discontinuation are shown in Figure 2. The patient continues to be monitored, at gradually increasing intervals, monitoring the residual foveal EZ changes (Figure 3). A timeline from diagnosis to discharge and follow‐up is shown in Figure 4.

## 3. Discussion

To the best of our knowledge, this is the first published report of PIP/TAZ‐associated bilateral optic disc edema accompanied with intraretinal and subretinal fluid. The patient experienced immediate improvements in her ocular symptoms after drug discontinuation, and there was gradual resolution of both clinical and OCT findings, except for the remaining foveal EZ changes, during the following 2 months. The EZ during an OCT scan is a bright reflective band in the outer retina that corresponds to the inner segments of the retinal photoreceptors. Disrupted or fragmented EZ suggests photoreceptor damage and often correlates with reduced visual acuity or metamorphopsia [1, 2].

The molecule of PIP/TAZ combines PIP (a fourth generation, extended‐spectrum penicillin) with TAZ (a beta‐lactamase inhibitor) providing a strong combination with broad activity against most gram‐positive and gram‐negative aerobic and anaerobic bacteria (including many pathogens producing beta‐lactamases) for the treatment of moderate‐to‐severe bacterial infections [16–18]. It has shown significant efficacy for the treatment of patients experiencing several body infections including (i) gynecological, (ii) skin/soft tissue, (iii) bone, (iv) lower respiratory tract, (v) intra‐abdominal, (vi) urinary tract, and (vii) neutropenic fever [16, 17]. The usual proposed therapeutic schedule is 3 to 4.5 g of PIP with 0.375 to 0.5 g of TAZ every eight to 6 h for 7 to 14 days.

The most common side effects after its administration include diarrhea (with or without *Clostridioides difficile* infection), dizziness, headaches, nausea, systemic allergic reactions/allergic shock, and skin and soft tissue reactions (including Stevens Johnson syndrome, toxic epidermal necrolysis, acute generalized exanthematous pustulosis, and drug reaction with eosinophilia and systemic symptoms [DRESS] syndrome) [19–21]. Other adverse events reported are mainly (i) hematological abnormalities and in particular neutropenia, hemolytic anemia, thrombocytopenia, and coagulopathy. Hemolytic anemia and thrombocytopenia were suggested to be immune‐mediated, while neutropenia was thought to be related to bone marrow suppression, (ii) liver injury, (iii) paresthesia, and (iv) drug‐induced fever [22–28]. Its combination with vancomycin was shown to increase the odds of acute kidney injury over vancomycin monotherapy, vancomycin plus cefepime and carbapenem, or PIP/TAZ monotherapy [29, 30].

Ocular adverse reactions after the administration of PIP/TAZ have been rarely reported. Photophobia and conjunctivitis are the two main ocular adverse effects reported in the prescribing information of the drug [31]. Although PIP/TAZ is not a recognizable cause of optic disc edema and/or retinal fluid accumulation, the close temporal association between drug exposure and symptom onset, the bilaterality, and the immediate improvement 24 h after its cessation support an association, which is characterized as possible after applying the Naranjo Adverse Drug Reaction Probability Scale. The mechanisms underlying this rare side effect are rather intriguing, since the drug has poor intraocular penetration when administered systemically compared to other antibiotics (such as moxifloxacin, carbapenems, and linezolid); therefore, it is given intravitreally for the treatment of ocular infections [32, 33]. When injected intravitreally to patients with endophthalmitis (in the dosage of 250 μg/0.1 mL), PIP/TAZ has shown good safety profile without any significant intraocular side effects [34–36].

The pathophysiological mechanisms underlying this possible rare side effect are unknown. However, several hypotheses can be made: (i) a transient disruption of the blood‐retinal barrier (BRB), leading to increased vascular permeability and intraretinal/subretinal fluid accumulation. This mechanism gains more interest considering the high sodium content of PIP/TAZ, which is the main reason of its cautious use in patients with decreased renal, hepatic, and/or cardiac function [31, 37]. One bottle of powder for solution for infusion of PIP/TAZ 4.5 g contains 217 mg of sodium [31]. Furthermore, PIP/TAZ was diluted in 100 mL of 0.9% sodium chloride in our patient, which contributed to an additional 900 mg of sodium chloride per dose. Given the fact that medical conditions such as sepsis, hypoxia, ischemia, and/or other idiosyncratic reactions can disturb normal BRB and increase vascular permeability for several molecules, PIP/TAZ could penetrate more easily into the posterior ocular space promoting osmotic attraction and further increasing fluid accumulation [38–40]. (ii) Although our patient did not experience any other common symptoms of ICP, such as headache, dizziness, pulsatile tinnitus, and nausea, a drug‐induced intracranial hypertension‐like process cannot be excluded. PIP/TAZ has not been classically associated with intracranial hypertension. However, several drug‐related alterations (including antimicrobial agents) in CSF dynamics have been reported [6, 7]. ICP may occur despite normal MRI imaging. LP with measurement of CSFOP is the diagnostic gold standard. In the present case, CSF evaluation could not be performed, due to the constant refusal of our patient, a fact that is being recognized as a major limitation of this report, and (iii) an immune‐mediated microvascular dysfunction. This possible mechanism is less likely given the absence of systemic manifestations and the significant improvement of our patient without any specific treatment and (iv) toxic or inflammatory effects of PIP/TAZ in the BRB. Drug‐induced retinal edema and serous detachments have been reported with the systematic administration of various drugs through several mechanisms involving endothelial dysfunction and breakdown of the retinal structure [41–43].

Other probable causes for the bilateral optic disc edema with retinal fluid found in our patient are (i) sepsis‐related vascular permeability. We consider this diagnosis less possible because when her ocular symptoms occurred, both the infection and her clinical status were markedly improved, and (ii) cerebral venous sinus thrombosis. Although we did not perform magnetic resonance venography, the absence of associated symptomatology and her rapid improvement without any specific treatment do not support this diagnosis and (iii) idiopathic intracranial hypertension following childbirth. The absence of headache, which characterizes this condition, as well as her rapid improvement without any further therapeutic approaches, lowers the possibility for this diagnosis [5].

## 4. Conclusion

Bilateral optic disc edema with serous retinal fluid after PIP/TAZ therapy is very rare. Clinicians should remain vigilant that optic disc edema with associated retinal fluid may occur during PIP/TAZ administration. Prompt discontinuation of the medication and ophthalmic evaluation should be immediately considered when visual symptoms arise. Further pharmacovigilance reporting is warranted to clarify the rarity of this adverse effect during PIP/TAZ administration.

## Funding

No funds were received.

## Disclosure

This work has not been deposited as a preprint and has not been previously presented at any scientific conference or seminar. All authors read and approved the final version of this manuscript.

## Ethics Statement

Ethical approval was not required for this single‐patient case report, in accordance with institutional and national regulations, as the report does not contain identifiable personal information.

## Consent

Informed written consent was obtained from the patient for publication of this manuscript, including images or other personal or clinical details of the patient.

## Conflicts of Interest

The authors declare no conflicts of interest.

## Supporting Information

Additional supporting information can be found online in the Supporting Information section.

## Supporting information


**Supporting Information** CARE check list.

## Data Availability

The data that support the findings of this study are available from the corresponding author upon reasonable request.
