# Supplementary material for: Piperacillin/Tazobactam‐Induced Bilateral Optic Disc Edema With Serous Retinal Fluid in a Young Female With Pelvic Abscess and Sepsis
Source: Case Rep Infect Dis. 2026 Jul 13;2026:2925958. doi: 10.1155/crdi/2925958 (PMC13359116; doi:10.1155/crdi/2925958)
Supplement: Supplementary file 1 — Supporting Information CARE check list. [file CRDI-2026-2925958-s001.docx]

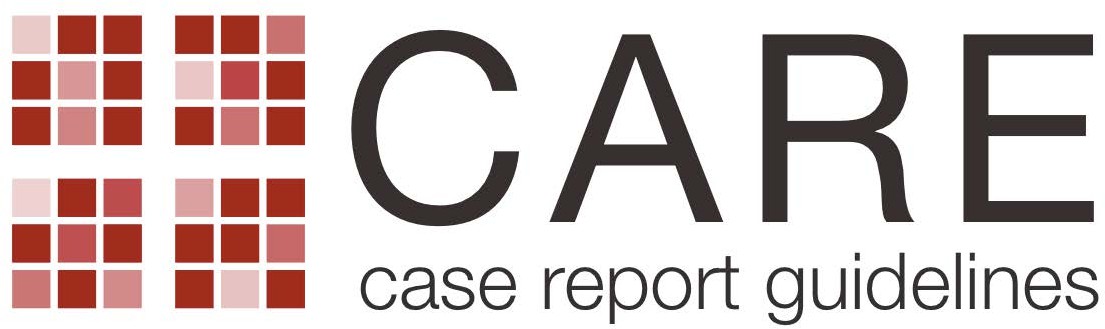
CARE Checklist of information to include when writing a case report
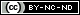


**Topic Item Checklist item description Reported on Line**

**Title 1** The diagnosis or intervention of primary focus followed by the words “case report” . . . . . . . . . . . . . . . . . . 1-3

**Key Words 2** 2 to 5 key words that identify diagnoses or interventions in this case report, including "case report" 55-56

**Abstract**

**(no references)**

**3a** Introduction: What is unique about this case and what does it add to the scientific literature? 32-38

**3b** Main symptoms and/or important clinical findings . . . . . . . . . . . . . . . . . . . . . . . . . . . . . . . . . . . . . . . . . . . . . . . . . . . 41-46

**3c** The main diagnoses, therapeutic interventions, and outcomes 48

**3d** Conclusion—What is the main “take-away” lesson(s) from this case? 49-51

**Introduction 4** One or two paragraphs summarizing why this case is unique (**may include** reference**s**) 61-79

**Patient Information 5a** De-identified patient specific information 82-90

**5b** Primary concerns and symptoms of the patient 83-84

**5c** Medical, family, and psycho-social history including relevant genetic information 87-90

**5d** Relevant past interventions with outcomes 82-83

**Clinical Findings**

**Timeline**

**Diagnostic Assessment**

**Therapeutic Intervention**

**Follow-up and Outcomes**

1. Describe significant physical examination (PE) and important clinical findings 91-96
2. Historical and current information from this episode of care organized as a timeline Figure4

**8a** Diagnostic testing (such as PE, laboratory testing, imaging, surveys). 101-104

**8b** Diagnostic challenges (such as access to testing, financial, or cultural) 105-107

**8c** Diagnosis (including other diagnoses considered) 113-133

**8d** Prognosis (such as staging in oncology) where applicable 133-135

**9a** Types of therapeutic intervention (such as pharmacologic, surgical, preventive, self-care) . . . . . . . . . . . . . . . . . 133-135

**9b** Administration of therapeutic intervention (such as dosage, strength, duration) 133-135

**9c** Changes in therapeutic intervention (with rationale) 133-135 (Figure 4)

**10a** Clinician and patient-assessed outcomes (if available) 113-133

**10b** Important follow-up diagnostic and other test results 113-133

**10c** Intervention adherence and tolerability (How was this assessed?) 113-133

**10d** Adverse and unanticipated events 113-133

**Discussion 11a** A scientific discussion of the strengths AND limitations associated with this case report 138-142,195-196

**11b** Discussion of the relevant medical literature **with references** 146-210

**11c** The scientific rationale for any conclusions (including assessment of possible causes) 213-217

**11d** The primary “take-away” lessons of this case report (without references) in a one paragraph conclusion 213-217

**Patient Perspective 12** The patient should share their perspective in one to two paragraphs on the treatment(s) they received . . . . 213-217

**Informed Consent 13** Did the patient give informed consent? Please provide if requested . . . . . . . . . . . . . . . . . . . . . . . . . . . . . . . . . . . . . . **Yes
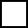
** **No
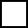
**
